# Invariant-Based Inverse Engineering for Fast and Robust Load Transport in a Double Pendulum Bridge Crane

**DOI:** 10.3390/e22030350

**Published:** 2020-03-18

**Authors:** Ion Lizuain, Ander Tobalina, Alvaro Rodriguez-Prieto, Juan Gonzalo Muga

**Affiliations:** 1 Department of Applied Mathematics, University of the Basque Country UPV/EHU, 48013 Bilbao, Spain; 2 Department of Physical Chemistry, University of the Basque Country UPV/EHU, 48940 Leioa, Spain

**Keywords:** shortcuts to adiabaticity, invariant-based engineering, mechatronics

## Abstract

We set a shortcut-to-adiabaticity strategy to design the trolley motion in a double-pendulum bridge crane. The trajectories found guarantee payload transport without residual excitation regardless of the initial conditions within the small oscillations regime. The results are compared with exact dynamics to set the working domain of the approach. The method is free from instabilities due to boundary effects or to resonances with the two natural frequencies.

## 1. Introduction

The concept of adiabaticity is ubiquitous in physics, but it is not fully exploited in mechanical engineering and control applications. Adiabatic theorems set the existence of approximate adiabatic invariants, such as the action integral in classical mechanics, when the control parameters of a given physical system vary slowly enough in time [1].

Adiabaticity is often used to drive systems in a robust manner. An example is a load hanging as a simple pendulum from a moving trolley on a bridge crane. If the trolley travels slowly enough between two points, the energy of the pendulum is an adiabatic invariant and stays constant along different smooth trolley trajectories for the same initial and final points. In particular, the minimum energy configuration, in which the oscillating mass stays at relative rest with respect to the suspension point, is preserved. More generally, for other initial states the final energy will not suffer excitations. However, the intrinsic slowness of such processes may be problematic, either because long operation times are impractical, or because during a long process time the ideal dynamics can be affected by the accumulation of random and/or uncontrollable perturbations that spoil the desired result.

To overcome these problems, “Shortcuts To Adiabaticity” (STA) methods have been developed in the last decade. The idea is to reach the same results of an adiabatic protocol in short times [2,3]. In STA, the adiabatic invariant is not kept constant throughout the process, but the initial value is recovered at final time. For the simple example of the load hanging from a moving trolley, the shortcuts are certain special and fast driving trajectories of the trolley that induce transitory excitations, but leave the load at final time with the same energy it had initially.

**Figure 1:**
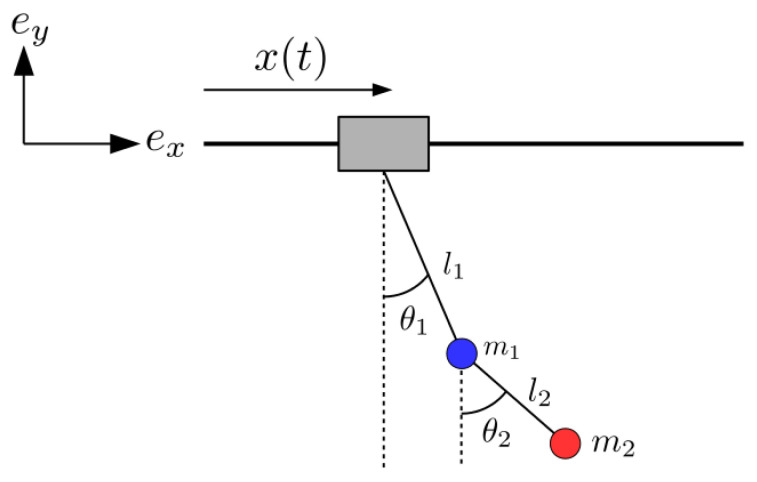
Double pendulum overhead crane scheme and relevant physical parameters.

STA methods have been succesfully applied to many different fields and processes in quantum systems, such as quantum computation [4,5,6,7], cooling [8], quantum transport [9,10], quantum state preparation [11,12,13,14], manipulation of cold atoms [15,16,17,18,19,20] or control of polyatomic molecules [21]. They have been also applied to design optical devices [22,23], and recently in mechanical engineering to design fast and robust protocols to control overhead cranes [24,25]. Perhaps surprisingly, because of the differing orders of magnitude involved, the physics of crane control are much related, in some formal aspects and domains even identical, to the physics of microscopic particle transport in moving traps [9]. In both domains the linearized models imply a moving harmonic oscillator. When the setting is more realistic though, beyond the simplest scenarios, the models become specific and require specialized treatment as in the system addressed in this paper, see Figure 1, a planar, double-pendulum, hook ($m_{1}$) and load ($m_{2}$) system suspended from a moving trolley. This is a relevant model as cranes behave like moving double pendulums due to different reasons, for example the large scale of the payload, or weighty hooks [26,27]. The control of a moving double pendulum dynamics is significantly more difficult than the single pendulum, with two unactuated degrees of freedom (angles $\theta_{1}$ and $\theta_{2}$ in Figure 1) and only one actuator (the trolley position *x*). Compared to studies on single pendulum cranes, this system is much less explored, for a recent brief review on recent papers and approaches applied see Reference [27]. Control approaches with and without feedback have been worked out and their pros and cons have been well discussed [26,27,28]. Our STA approach is presented here in an elementary way without feedback but it may be adapted and incorporated into methods with feedback as well.

Among the different STA approaches, dynamical invariant based inverse engineering is one of the most successful and is the one followed here. The essence of the method is to identify exact (rather than adiabatic) dynamical invariants, set boundary conditions to cancel final excitation, design the dynamics compatible with these conditions, and deduce the necessary controls from the dynamics thanks to the relations between dynamical invariants and Hamiltonian. For the moving double pendulum the STA consists on designing trolley trajectories x(t) from x=0 to x=d so that the system ends up at final process time $t_{f}$ without excitations. From the point of view of STA process design, this system poses interesting, non-trivial challenges with respect to the single pendulum, as we shall see.

The article is organized as follows. The physical model and Hamiltonian of the system are set in Section 2, both in exact form and in the small oscillation regime. Dynamically decoupled normal modes are found in Section 3, and then the STA protocol is designed in Section 4. Numerical results are presented in Section 5 and, finally, in Section 6 we end with the conclusions and discuss some open questions.

## 2. Physical Model

The physical model and relevant parameters are shown in Figure 1. The model assumes several conditions and idealizations: (i) the mass of the wires and friction are neglected; (ii) point masses; (iii) constant wire lengths $l_{1}$ and $l_{2}$; (iv) the trolley position is treated as a control parameter rather than a dynamical variable. This last assumption is a common and simplifying assumption [29] that requires a good controller, but a more fundamental approach considering the trolley as a dynamical variable is also possible as in Reference [24].

### 2.1. Lagrangian

In terms of the angles $\theta_{1}$ and $\theta_{2}$, see Figure 1, the Cartesian coordinates of each mass in a rest frame are given by

(1) $$\begin{array}{ccc} x_{1}=x+l_{1}\sin\theta_{1}, & \; & y_{1}=-l_{1}\cos\theta_{1},\\ x_{2}=x_{1}+l_{2}\sin\theta_{2}, & \; & y_{2}=y_{1}-l_{2}\cos\theta_{2}, \end{array}$$

so kinetic (T) and potential (V) energies are given by

(2) $$\begin{array}{ccc} T & = & \frac{1}{2}m_{1}\left({\dot{x}}_{1}^{2}+{\dot{y}}_{1}^{2}\right)+\frac{1}{2}m_{2}\left({\dot{x}}_{2}^{2}+{\dot{y}}_{2}^{2}\right),\;V=m_{1}gy_{1}+m_{2}gy_{2}, \end{array}$$

where the dots represent time derivatives. The Lagrangian of the system using $\theta_{1}$ and $\theta_{2}$ as generalized coordinates and dynamical variables and x(t) as a control parameter will be given by

(3) $$\begin{array}{ccc} L & = & L(\theta_{i},{\dot{\theta }}_{i};t)=T-V. \end{array}$$

To avoid deformations or excessive tensions, cranes usually work in the small oscillations regime, in which $\theta_{i}$ are small so that we may approximate $\sin\theta_{i}\approx \theta_{i}$ and $\cos\theta_{i}\approx 1-\theta_{i}^{2}/2$. Angular velocities $\dot{\theta }$ will be considered small as well. This approximation linearizes the dynamical equations of motion of the system. Results found with exact and approximate dynamics will be compared later to check the validity of the approximation and its limits.

In this small oscillation regime and keeping up to second order quadratic terms in $\theta_{i}$ and ${\dot{\theta }}_{i}$, kinetic and potential energies are given by

(4) $$\begin{array}{ccc} T & \approx & \frac{1}{2}M{\dot{x}}^{2}+\frac{1}{2}\left({\dot{\theta }}_{1},{\dot{\theta }}_{2}\right)\left(\begin{array}{cc} Ml_{1}^{2} & m_{2}l_{1}l_{2}\\ m_{2}l_{1}l_{2} & m_{2}l_{2}^{2} \end{array}\right)\left(\begin{array}{c} {\dot{\theta }}_{1}\\ {\dot{\theta }}_{2} \end{array}\right)+\dot{x}\left(Ml_{1},m_{2}l_{2}\right)\left(\begin{array}{c} {\dot{\theta }}_{1}\\ {\dot{\theta }}_{2} \end{array}\right),\\ V & \approx & -Mgl_{1}\left(1-\frac{\theta_{1}^{2}}{2}\right)-m_{2}gl_{2}\left(1-\frac{\theta_{2}^{2}}{2}\right), \end{array}$$

where *M* denotes the total mass $M=m_{1}+m_{2}$. The Lagrangian becomes

(5) $$\begin{array}{ccc} L\approx T-V & = & \frac{1}{2}\left({\dot{\theta }}_{1},{\dot{\theta }}_{2}\right)\left(\begin{array}{cc} Ml_{1}^{2} & m_{2}l_{1}l_{2}\\ m_{2}l_{1}l_{2} & m_{2}l_{2}^{2} \end{array}\right)\left(\begin{array}{c} {\dot{\theta }}_{1}\\ {\dot{\theta }}_{2} \end{array}\right)-\frac{1}{2}\left(\theta_{1},\theta_{2}\right)\left(\begin{array}{cc} Mgl_{1} & 0\\ 0 & m_{2}gl_{2} \end{array}\right)\left(\begin{array}{c} \theta_{1}\\ \theta_{2} \end{array}\right)\\ & + & \dot{x}\left(Ml_{1},m_{2}l_{2}\right)\left(\begin{array}{c} {\dot{\theta }}_{1}\\ {\dot{\theta }}_{2} \end{array}\right), \end{array}$$

where purely time-dependent and constant terms have been omitted since they do not affect the dynamics.

### 2.2. Hamiltonian

To implement a Hamiltonian formulation, which is more convenient to treat the invariants and inverse engineering of trolley trajectories, we need the conjugate momentum of each $\theta_{i}$,

(6) $$\begin{array}{ccc} p_{\theta_{1}} & = & \frac{\partial L_{\theta }}{\partial {\dot{\theta }}_{1}}=Ml_{1}\dot{x}+Ml_{1}^{2}{\dot{\theta }}_{1}+m_{2}l_{1}l_{2}{\dot{\theta }}_{2},\\ p_{\theta_{2}} & = & \frac{\partial L_{\theta }}{\partial {\dot{\theta }}_{2}}=m_{2}l_{2}\dot{x}+m_{2}l_{1}l_{2}{\dot{\theta }}_{1}+m_{2}l_{2}^{2}{\dot{\theta }}_{2}. \end{array}$$

These relations can be inverted to have the generalized velocities ${\dot{\theta }}_{i}$ in terms of the generalized momenta $p_{\theta_{i}}$. The Hamiltonian is found from the Lagrangian as

(7) $$\begin{array}{ccc} H_{\theta } & = & \sum_{i=1}^{2}{\dot{\theta }}_{i}p_{\theta_{i}}-L=\frac{p_{\theta_{1}}^{2}}{2m_{1}l_{1}^{2}}+\frac{p_{\theta_{2}}^{2}}{2\mu l_{2}^{2}}-\frac{p_{\theta_{1}}p_{\theta_{2}}}{m_{1}l_{1}l_{2}}+\frac{1}{2}Mgl_{1}\theta_{1}^{2}+\frac{1}{2}m_{2}gl_{2}\theta_{2}^{2}-\dot{x}\frac{p_{\theta_{1}}}{l_{1}}, \end{array}$$

where $\mu =\frac{m_{1}m_{2}}{m_{1}+m_{2}}$ is the reduced mass and where constant terms that do not affect the dynamics have been neglected. In matrix representation, this Hamiltonian can be written as

(8) $$\begin{array}{ccc} H_{\theta } & = & \frac{1}{2}\left(p_{\theta_{1}},p_{\theta_{2}}\right)T\left(\begin{array}{c} p_{\theta_{1}}\\ p_{\theta_{2}} \end{array}\right)+\frac{1}{2}\left(\theta_{1},\theta_{2}\right)K\left(\begin{array}{c} \theta_{1}\\ \theta_{2} \end{array}\right)-\left(\frac{\dot{x}}{l_{1}},0\right)\left(\begin{array}{c} p_{\theta_{1}}\\ p_{\theta_{2}} \end{array}\right), \end{array}$$

where

$$\;T=\left(\begin{array}{cc} \frac{1}{m_{1}l_{1}^{2}} & \frac{-1}{m_{1}l_{1}l_{2}}\\ \frac{-1}{m_{1}l_{1}l_{2}} & \frac{m_{1}+m_{2}}{m_{1}m_{2}l_{2}^{2}} \end{array}\right)\;;K=\left(\begin{array}{cc} Mgl_{1} & 0\\ 0 & m_{2}gl_{2} \end{array}\right).$$

Whereas the potential matrix *K* is diagonal, the kinetic matrix *T* is not, i. e., $p_{\theta_{1}}$ and $p_{\theta_{2}}$ momenta are coupled. We want to find a coordinate transformation, i. e., normal modes, where both the coordinates and momenta are uncoupled so that we can easily get the dynamical invariants to inverse engineer x(t). In the following section, these normal modes will be calculated following Reference [30]. Normal modes for the double pendulum with fixed suspension point are known [31], but our treatment takes the motion of the trolley into account. Finding dynamical normal modes for quadratic *time-dependent* Hamiltonians is generically non-trivial [30], but in this system the task is facilitated by the fact that the time-dependence appears in linear terms via $\dot{x}\left(t\right)$.

## 3. Normal Modes

### 3.1. Diagonalization of $H_{\theta }$

Let us first define a new set of coordinates $u_{1}$ and $u_{2}$ by the linear transformation

(9) $$\left(\begin{array}{c} u_{1}\\ u_{2} \end{array}\right)=A\left(\begin{array}{c} \theta_{1}\\ \theta_{2} \end{array}\right),$$

where the *A* matrix is yet to be determined. The corresponding momenta transform according to Reference [30]

(10) $$\left(\begin{array}{c} p_{u_{1}}\\ p_{u_{2}} \end{array}\right)=A^{-T}\left(\begin{array}{c} p_{\theta_{1}}\\ p_{\theta_{2}} \end{array}\right),$$

where $A^{-T}={\left(A^{-1}\right)}^{T}$ stands for the transpose of the inverse matrix. The Hamiltonian in these variables reads

(11) $$\begin{array}{ccc} H_{u} & = & \frac{1}{2}\left(p_{u_{1}},p_{u_{2}}\right)\left(ATA^{T}\right)\left(\begin{array}{c} p_{u_{1}}\\ p_{u_{2}} \end{array}\right)+\frac{1}{2}\left(u_{1},u_{2}\right)\left(A^{-T}KA^{-1}\right)\left(\begin{array}{c} u_{1}\\ u_{2} \end{array}\right)-\left(\frac{\dot{x}}{l_{1}},0\right)A^{T}\left(\begin{array}{c} p_{u_{1}}\\ p_{u_{2}} \end{array}\right) \end{array}$$

We now look for a transformation matrix *A* that diagonalizes simultaneously both the $ATA^{T}$ and $A^{-T}KA^{-1}$ matrices in the expression above. To do so it is useful to define the following matrix

(12) $$\tilde{T}=K^{1/2}TK^{1/2},$$

which is symmetric and positive definite and therefore can be diagonalized by an orthogonal matrix O. Without loss of generality, this orthogonal matrix O can be parametrized as

(13) $$O=\left(\begin{array}{cc} \cos\theta & -\sin\theta \\ \sin\theta & \cos\theta \end{array}\right),$$

and choosing the parameter θ (not to be confused with the angles $\theta_{i}$) by

(14) $$\tan2\theta =\frac{2l_{1}}{\left(l_{1}-l_{2}\right)}\sqrt{\frac{m_{2}l_{2}}{Ml_{1}}},$$

we have that

(15) $$O^{T}\tilde{T}O=\mathrm{diag}\left(\omega_{1}^{2},\omega_{2}^{2}\right)=T_{d}.$$

The eigenvalues $\omega_{i}^{2}$ are positive since $\tilde{T}$ is a positive definite matrix, and have dimensions of (angular) frequency square. The explicit expressions are

$$\omega_{1}^{2}=\frac{g}{m_{1}l_{1}l_{2}}\left(-\sqrt{Mm_{2}l_{1}l_{2}}\sin2\theta +Ml_{1}\sin^{2}\theta +Ml_{2}\cos^{2}\theta \right),$$

$$\;\omega_{2}^{2}=\frac{g}{m_{1}l_{1}l_{2}}\left(\sqrt{Mm_{2}l_{1}l_{2}}\sin2\theta +Ml_{1}\cos^{2}\theta +Ml_{2}\sin^{2}\theta \right),$$

in agreement with the eigenfrequencies given in Reference [31]. Now, by writing the transformation matrix as

(16) $$\begin{array}{ccc} \;A & = & O^{T}K^{1/2}=\left(\begin{array}{cc} \sqrt{Mgl_{1}}\cos\theta & \sqrt{m_{2}gl_{2}}\sin\theta \\ -\sqrt{Mgl_{1}}\sin\theta & \sqrt{m_{2}gl_{2}}\cos\theta \end{array}\right)\;, \end{array}$$

both quadratic terms in the transformed Hamiltonian (11) are diagonal since

(17) $$\begin{array}{ccc} ATA^{T} & = & O^{T}K^{1/2}TK^{1/2}O=O^{T}\tilde{T}O=T_{d}, \end{array}$$

(18) $$\begin{array}{ccc} A^{-T}KA^{-1} & = & O^{T}K^{-1/2}KK^{-1/2}O=1. \end{array}$$

Finally, the Hamiltonian (11) takes the uncoupled form

(19) $$\begin{array}{ccc} H_{u} & = & \frac{1}{2}\sum_{i=1}^{2}\left(\omega_{i}^{2}p_{u_{i}}^{2}+u_{i}^{2}\right)+\dot{x}\sqrt{\frac{Mg}{l_{1}}}\left(-p_{u_{1}}\cos\theta +p_{u_{2}}\sin\theta \right). \end{array}$$

### 3.2. Lewis-Leach Family of Hamiltonians and Second Canonical Transformation

The Lewis-Leach (LL) family of Hamiltonians are of the form [32]

(20) $$H_{LL}=\frac{1}{2}\left[p^{2}+\Omega \left(t\right)q^{2}\right]-F\left(t\right)q,$$

i.e., quadratic Hamiltonians with linear in position terms. For them quadratic invariants are explicitly known. By a suitable canonical transformation to some generalized coordinates $\left\{q_{i},p_{i}\right\}$, we shall transform $H_{u}$ into this form. This can be easily achieved just by exchanging momentum and coordinate [33]. The transformation is generated by $F_{1}=u_{1}q_{1}+u_{2}q_{2}$ which gives the new coordinates and momenta in terms of the old ones as follows,

(21) $$\begin{array}{c} p_{u_{i}}=\frac{\partial F_{1}}{\partial u_{i}}=q_{i},\;p_{i}=-\frac{\partial F_{1}}{\partial q_{i}}=-u_{i}. \end{array}$$

By using this canonical transformation, the new Hamiltonian is

(22) $$\begin{array}{ccc} H_{q} & = & H_{1}+H_{2}, \end{array}$$

a sum of two independent forced harmonic oscillators that belong to the LL family,

(23) $$\begin{array}{ccc} H_{1} & = & \frac{1}{2}\left(p_{1}^{2}+\omega_{1}^{2}q_{1}^{2}\right)-q_{1}\dot{x}\sqrt{\frac{Mg}{l_{1}}}\cos\theta ,\\ H_{2} & = & \frac{1}{2}\left(p_{2}^{2}+\omega_{2}^{2}q_{2}^{2}\right)+q_{2}\dot{x}\sqrt{\frac{Mg}{l_{1}}}\sin\theta . \end{array}$$

### 3.3. Explicit Expression of Normal Mode Coordinates

Taking into account the two canonical transformations, the explicit expression of the normal mode coordinates and momenta $\left\{q_{i},p_{i}\right\}$ in terms of the original variables $\left\{\theta_{i},p_{\theta_{i}}\right\}$ is

(24) $$\begin{array}{ccc} \left(\begin{array}{c} q_{1}\\ q_{2}\\ p_{1}\\ p_{2} \end{array}\right) & = & \left(\begin{array}{cc} 0 & I_{2}\\ -I_{2} & 0 \end{array}\right)\left(\begin{array}{cc} A & 0\\ 0 & A^{-T} \end{array}\right)\left(\begin{array}{c} \theta_{1}\\ \theta_{2}\\ p_{\theta_{1}}\\ p_{\theta_{2}} \end{array}\right), \end{array}$$

where $I_{2}$ is the 2×2 identity matrix and, using the explicit expression of *A* in (16), we have

(25) $$\begin{array}{ccc} q_{1} & = & \frac{\cos\theta }{\sqrt{Mgl_{1}}}p_{\theta_{1}}+\frac{\sin\theta }{\sqrt{m_{2}gl_{2}}}p_{\theta_{2}},\\ q_{2} & = & -\frac{\sin\theta }{\sqrt{Mgl_{1}}}p_{\theta_{1}}+\frac{\cos\theta }{\sqrt{m_{2}gl_{2}}}p_{\theta_{2}},\\ p_{1} & = & -\cos\theta \sqrt{Mgl_{1}}\theta_{1}-\sin\theta \sqrt{m_{2}gl_{2}}\theta_{2},\\ p_{2} & = & \sin\theta \sqrt{Mgl_{1}}\theta_{1}-\cos\theta \sqrt{m_{2}gl_{2}}\theta_{2}. \end{array}$$

## 4. Designing the STA Protocol

We are now ready to define the invariants and design the driving function x(t).

### 4.1. Dynamical Invariants

A dynamical invariant of a Hamiltonian system remains constant during the time evolution [34]. Labelling the dynamical invariant of the Hamiltonian $H_{i}$ as $I_{i}$ we have that

(26) $$\begin{array}{ccc} \frac{dI_{i}}{dt} & = & \partial_{t}I_{i}+\left\{I_{i},H_{i}\right\}=0, \end{array}$$

with $\left\{I_{i},H_{i}\right\}$ being the Poisson bracket. The sum of invariants $I=I_{1}+I_{2}$ is invariant with respect to the sum of Hamiltonians $H_{q}=H_{1}+H_{2}$ since

$$\begin{array}{ccc} \frac{dI}{dt} & = & \left\{I,H_{q}\right\}+\partial_{t}I=\left\{I_{1}+I_{2},H_{1}+H_{2}\right\}+\partial_{t}\left(I_{1}+I_{2}\right)\\ & = & \left(\left\{I_{1},H_{1}\right\}+\partial_{t}I_{1}\right)+\left(\left\{I_{2},H_{2}\right\}+\partial_{t}I_{2}\right)+\left\{I_{1},H_{2}\right\}+\left\{I_{2},H_{1}\right\}=0. \end{array}$$

The invariants for (23) have the explicit form [32]

(27) $$\begin{array}{ccc} I_{i} & = & \frac{1}{2}{\left(p_{i}-{\dot{\alpha }}_{i}\right)}^{2}+\frac{\omega_{i}^{2}}{2}{\left(q_{i}-\alpha_{i}\right)}^{2}, \end{array}$$

provided the functions $\alpha_{i}$ satisfy the following Newton equations,

(28) $$\begin{array}{ccc} {\ddot{\alpha }}_{1}+\omega_{1}^{2}\alpha_{1} & = & \dot{x}\sqrt{\frac{Mg}{l_{1}}}\cos\theta , \end{array}$$

(29) $$\begin{array}{ccc} {\ddot{\alpha }}_{2}+\omega_{2}^{2}\alpha_{2} & = & -\dot{x}\sqrt{\frac{Mg}{l_{1}}}\sin\theta . \end{array}$$

These $\alpha_{i}$ functions may be regarded as auxiliary, reference, special “displacements” in two forced harmonic oscillators. Let us underline that the actual motion for a specific transport process is described by the $q_{i}$ rather than by the $\alpha_{i}$ (which represent just a particular case of all possible $q_{i}$). Note by the way that the $q_{i}$ satisfy the same Newton equations (with the same forces) as the $\alpha_{i}$. However, we shall impose to $\alpha_{i}$ boundary conditions that will guarantee zero final excitations whereas the initial conditions for the $q_{i}$ are arbitrary.

### 4.2. Boundary Conditions (BC) for x(t) and $\alpha_{i}\left(t\right)$

We shall assume a transport from x(0)=0 to $x(t_{f})=d$ with additional smooth boundary conditions for the trolley velocity, $\dot{x}\left(t_{b}\right)=0$ for $t_{b}=0,t_{f}$. We shall further assume that the auxiliary functions $\alpha_{i}$, as well as their first and second time derivatives vanish at boundary times $t_{b}=0,t_{f}$. We therefore have in principle a total of sixteen boundary conditions (BC), namely

(30) $$\begin{array}{ccc} \alpha_{i}\left(t_{b}\right) & = & {\dot{\alpha }}_{i}\left(t_{b}\right)={\ddot{\alpha }}_{i}\left(t_{b}\right)=0,\\ x(0) & = & 0;x(t_{f})=d,\\ \dot{x}\left(0\right) & = & 0;\dot{x}\left(t_{f}\right)=0. \end{array}$$

These boundary conditions guarantee that each invariant $I_{i}$ coincides with the corresponding Hamiltonian $H_{i}$ at initial and final times, see (27),

$$\begin{array}{c} H_{q}\left(t_{b}\right)=H_{1}\left(t_{b}\right)+H_{2}\left(t_{b}\right)=I_{1}\left(t_{b}\right)+I_{2}\left(t_{b}\right)=I\left(t_{b}\right). \end{array}$$

At these boundary times, and due to the $\dot{x}\left(t_{b}\right)=0$ boundary condition, the Hamiltonian represents the total mechanical energy of the system, i. e. $E\left(t_{b}\right)=H_{q}\left(t_{b}\right)$. If a fast finite-time process is designed so that the auxiliary functions $\alpha_{i}$ satisfy the imposed boundary conditions, the energy at final and initial times -regardless of the initial conditions of the hook and load, that is, regardless of the initial conditions set for $q_{i}\left(0\right)$ and its derivatives- will coincide since

$$E\left(0\right)=H_{q}\left(0\right)=I\left(0\right)=I\left(t_{f}\right)=H_{q}\left(t_{f}\right)=E\left(t_{f}\right).$$

Note that in principle the only conditions needed to guarantee $I\left(t_{b}\right)=H\left(t_{b}\right)$ are the ones for $\alpha (t_{b})$ and $\dot{\alpha }\left(t_{b}\right)$. The others have a physical motivation as the desired boundaries for the trolley motion (on $x(t_{b})$ and $\dot{x}\left(t_{b}\right)$) or are a consequence of the former ones (the ones on $\ddot{\alpha }\left(t_{b}\right)$ because of (28) and (29)).

In the following subsection we will show how to construct the trolley trajectory x(t) so that the desired conditions in (30) are satisfied.

### 4.3. Inverse Engineering

We start by proposing the following ansatz for the trolley velocity $\dot{x}\left(t\right)$, symmetric with respect to $t_{f}/2$,

(31) $$\dot{x}\left(t\right)=\sum_{j=1}^{3}a_{j}\sin\frac{(2j-1)\pi t}{t_{f}},$$

with three free parameters $a_{1}$, $a_{2}$, and $a_{3}$ that will be determined from the following three conditions (the second line involves two conditions, one for each frequency, as justified in the Appendix):

(32) $$\begin{array}{ccc} \int_{0}^{t_{f}}\dot{x}\left(\tau \right)d\tau & = & d, \end{array}$$

(33) $$\begin{array}{ccc} \int_{0}^{t_{f}}\dot{x}\left(\tau \right)\cos\left[\omega_{j}\left(\tau -\frac{t_{f}}{2}\right)\right]d\tau & = & 0, \end{array}$$

for j=1,2.

Different functional forms are possible, but this ansatz is chosen for simplicity and because of very useful properties discussed in the Appendix A (it avoids resonance and boundary effects). It is also remarkable that an ansatz with only three free parameters satisfies the full set of sixteen boundary conditions in (30), see further details in the Appendix A.

The three free parameters can be therefore written in terms of the system physical parameters as

(34) $$\begin{array}{ccc} a_{1} & = & \frac{75\pi d\left(\omega_{1}^{2}t_{f}^{2}-\pi^{2}\right)\left(\omega_{2}^{2}t_{f}^{2}-\pi^{2}\right)}{128t_{f}^{5}\omega_{1}^{2}\omega_{2}^{2}}, \end{array}$$

(35) $$\begin{array}{ccc} a_{2} & = & -\frac{75\pi d\left(\omega_{1}^{2}t_{f}^{2}-9\pi^{2}\right)\left(\omega_{2}^{2}t_{f}^{2}-9\pi^{2}\right)}{256t_{f}^{5}\omega_{1}^{2}\omega_{2}^{2}}, \end{array}$$

(36) $$\begin{array}{ccc} a_{3} & = & \frac{15\pi d\left(\omega_{1}^{2}t_{f}^{2}-25\pi^{2}\right)\left(\omega_{2}^{2}t_{f}^{2}-25\pi^{2}\right)}{256t_{f}^{5}\omega_{1}^{2}\omega_{2}^{2}}. \end{array}$$

**Figure 2:**
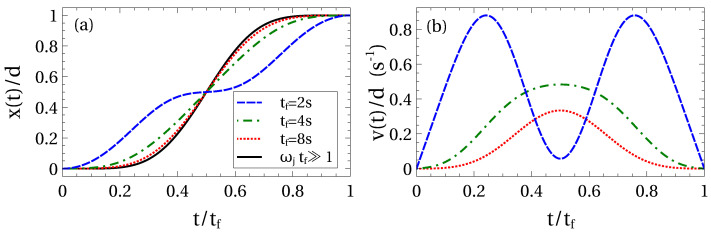
(Color online) Trolley trajectories x(t) and velocities $v\left(t\right)=\dot{x}\left(t\right)$ for different final times: $t_{f}=2s$ (blue-dashed line), $t_{f}=4s$ (green-dot-dashed line), $t_{f}=8s$ (red-dotted line). Compare to the “long time behaviour” in (a) of (38) (black-solid line). Other parameters are: $m_{1}=1$ kg, $m_{2}=0.5$ kg, $l_{1}=1$ m, $l_{2}=0.2$ m.

These parameters determine completely the velocity of the trolley by (31), and its trajectory is simply the integral

(37) $$\begin{array}{c} x\left(t\right)=\int_{0}^{t}\dot{x}\left(\tau \right)d\tau , \end{array}$$

which gives an explicit but lengthy expression. See some trolley trajectories and velocities in Figure 2. For long transport times ($\omega_{j}t_{f}\gg \pi$) the trolley trajectory becomes independent of the masses and lengths of the pendulum and tends to

(38) $$\begin{array}{ccc} x_{\infty }\left(t\right) & = & d\left[\frac{1}{2}-\frac{75}{128}\cos\left(\frac{\pi t}{t_{f}}\right)+\frac{25}{256}\cos\left(\frac{3\pi t}{t_{f}}\right)-\frac{3}{256}\cos\left(\frac{5\pi t}{t_{f}}\right)\right]. \end{array}$$

This trajectory implies a maximal velocity $v_{max}=\left(15\pi /16\right)\left(d/t_{f}\right)$ at $t=t_{f}/2$. In this asymptotic scenario there is only one acceleration time segment up to $t_{f}/2$ and a subsequent braking segment.

For short times compared to eigenperiods there are several segments of acceleration and braking. In any case this regime is less interesting in practice since the system deviates from the harmonic regime.

## 5. Numerical Results

### 5.1. Time Evolution of Suspension Angles

**Figure 3:**
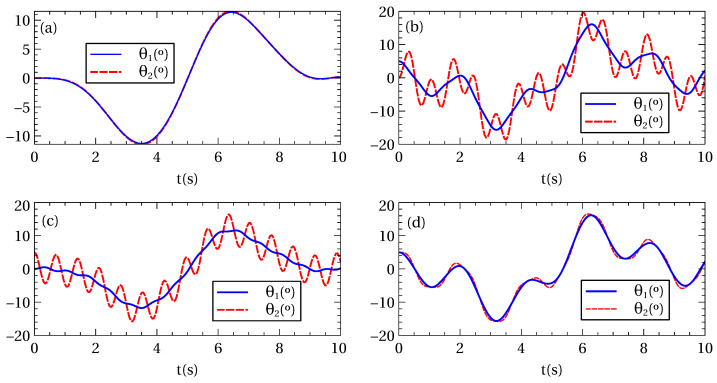
(Color online) Time evolution of the suspension angles for a transport of d=15 m in a time $t_{f}=10$ s. We have numerically integrated the exact dynamical equations using the exact Lagrangian (3) with different initial conditions: (**a**) $\theta_{1}\left(0\right)=0^{{}^{\circ}}$, $\theta_{2}\left(0\right)=0^{{}^{\circ}}$; (**b**) $\theta_{1}\left(0\right)=5^{{}^{\circ}}$, $\theta_{2}\left(0\right)=0^{{}^{\circ}}$; (**c**) $\theta_{1}\left(0\right)=0^{{}^{\circ}}$, $\theta_{2}\left(0\right)=5^{{}^{\circ}}$; and (**d**) $\theta_{1}\left(0\right)=5^{{}^{\circ}}$, $\theta_{2}\left(0\right)=5^{{}^{\circ}}$, with ${\dot{\theta }}_{1}\left(0\right)={\dot{\theta }}_{2}\left(0\right)=0$ in all cases. In the scale of the figure the results using the approximate Lagrangian (5) or the exact one are indistinguishable. Other parameters are: $m_{1}=1$ kg, $m_{2}=0.5$ kg, $l_{1}=1$ m, $l_{2}=0.2$ m.

Once the trolley trajectory is designed, the dynamical evolution of the system can be found by numerically integrating the Euler-Lagrange equations of motion using either the exact Lagrangian (3) or the approximate Lagrangian in the harmonic (small oscillations) approximation (5). In Figure 3 some examples of the time evolution of the suspension angles $\theta_{1}$ and $\theta_{2}$ during transport are shown. The initial and final angles are not equal (unless the system is initially at equilibrium), but this is not a requirement for ending with the initial energy. The calculation has been done using the exact Lagrangian, but the results are undistinguishable in the scale of the figure when using the approximate Lagrangian since the involved angles are small throughout the whole transport process. For larger transport distance *d* or smaller process time $t_{f}$ these differences will increase and will lead to some errors due to the anharmonicity of the exact model as will be discussed in the following section.

### 5.2. Anharmonic Effects

**Figure 4:**
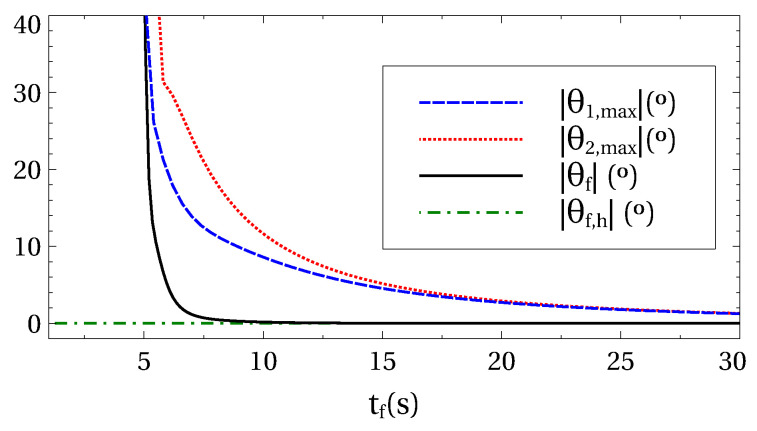
(Color online) Maximum swing angles during the process as a function of the duration $t_{f}$ (red dotted and blue dashed lines). For very rapid operations (small $t_{f}$), larger angles are involved and the harmonic approximation breaks down. Fictitious angle $\theta_{f}$ (black-solid line), which basically is a measure of the final excitation energy, see the main text, as a function of $t_{f}$. In the harmonic approximation this angle is zero by construction ($\theta_{f,h}$, green-dashed-dotted line). System assumed initially in equilibrium. Other parameters are: $m_{1}=30$ kg, $m_{2}=3$ kg, $l_{1}=30$ m, $l_{2}=3$ m, d=15 m. The natural periods of the modes are $T_{1}=2\pi /\omega_{1}=11.048$ s and $T_{2}=2\pi /\omega_{2}=3.298$ s.

For rapid transport operations, the involved angles are larger and the harmonic approximation breaks down, see Figure 4. Therefore, some deviations from the ideal results (i.e., equal final and initial energies) should be expected.

To quantify the excitation at final time in a way that is easy to understand and visualize, we measure the final energy ΔE in terms of a fictitious angle $\theta_{f}$. This angle is defined as follows: *(i)* the load and hook are initially in equilibrium (at rest in the vertical position); and *(ii)* the final energy is artificially interpreted as pure potential energy for a configuration where load and hook are at rest along a line with $\theta_{f}=\theta_{1}=\theta_{2}$. In other words: $\theta_{f}$ is the final angle when the final energy is considered to be purely potential and the two suspension angles coincide. Using (2) we may write

(39) $$\begin{array}{c} \Delta E=-2E_{0}\sin^{2}\frac{\theta_{f}}{2}, \end{array}$$

with $E_{0}=-m_{1}gl_{1}-m_{2}g\left(l_{1}+l_{2}\right)$ being the energy for the equilibrium configuration. In Figure 4, this fictitious angle is plotted as a function of the process duration time $t_{f}$.

### 5.3. Stability

**Figure 5:**
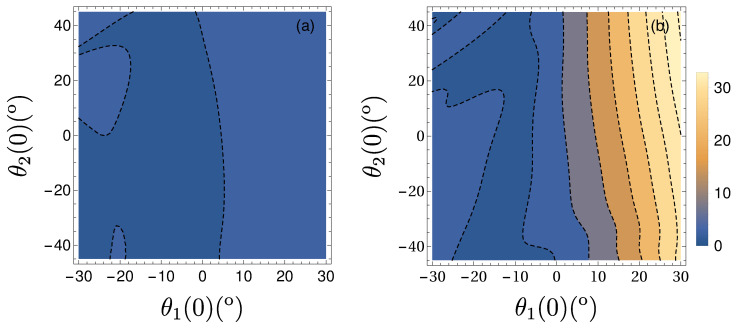
(Color online) Difference between final and initial energy measured by the modulus of the fictitious angle $\theta_{f}$ (in °) as a function of the deviations from equilibrium configuration of either $\theta_{1}\left(0\right)$ or $\theta_{2}\left(0\right)$ after solving the exact dynamics with Lagrangian (2). (**a**) Final fictitious angle for the inverse-engineered trolley trajectory (37). (**b**) Final fictitious angle for the postulated cubic trajectory (40). Other parameters are: $m_{1}=1$ kg, $m_{2}=0.5$ kg, $l_{1}=1$ m, $l_{2}=0.2$ m, d=15 m and $t_{f}=5$ s. The system is assummed initially at rest, ${\dot{\theta }}_{1}\left(0\right)={\dot{\theta }}_{2}\left(0\right)=0$.

The stability of the proposed transport protocol can be studied by allowing some initial deviations of the angles $\theta_{1}\left(0\right)$ or $\theta_{2}\left(0\right)$ from the equilibrium positions. In Figure 5a, the final time energy excitation, measured in units of the fictitious angle $\theta_{f}$ (39), is plotted as a function of these deviations.

We will compare the resulting excitation with that for a simple third order polynomial ansatz for the trolley trajectory,

(40) $$\begin{array}{c} x\left(t\right)=3d{\left(\frac{t}{t_{f}}\right)}^{2}-2d{\left(\frac{t}{t_{f}}\right)}^{3}, \end{array}$$

which satisfies the four BCs in (30) for x(t) but not those for the auxiliar functions $\alpha_{i}$. As shown in Figure 5b (which should be compared with Figure 5a), the excitation at final time using this simple trajectory is much larger that the one using the inverse engineered trajectory. Our inverse engineering method leads to much more robust results.

### 5.4. Example Limiting the Maximal Trolley Speed

The engine power and safety considerations imply limits to the trolley speed. In this example we test the effect of such a limit. We set a load $m_{2}=1000$ kg transported a distance d=40 m. We also set a hook mass $m_{1}=150$ kg, $l_{2}=5$ m, and $l_{1}=40$ m. A maximum velocity of 2 m/s is assumed.

**Figure 6:**
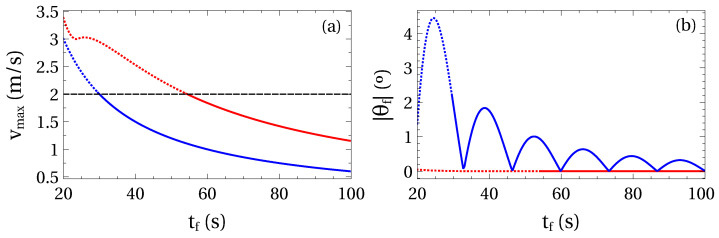
(Color online) Comparison of two transport protocols, the inverse engineered trolley trajectory (37) (red) and the cubic trajectory (40) (blue). The dotted line is for segments where the maximal trolley velocity is larger than 2 m/s, whereas in solid line segments the maximal velocity is below that value. (**a**) Maximum trolley velocity during the process and (**b**) excitation at final time measured by the fictitious angle $\theta_{f}$. Rest of parameters: $m_{1}=150$ kg, $m_{2}=1000$ kg, $l_{1}=40$ m, $l_{2}=5$ m and d=40 m. System initially at equilibrium. The natural periods of the modes are $T_{1}=2\pi /\omega_{1}=13.376$ s and $T_{2}=2\pi /\omega_{2}=1.538$ s.

With this data, two transport protocols are compared in Figure 6: (i) inverse engineered trolley trajectory (37) and (ii) directly postulated cubic trajectory (40). For initial conditions at equilibrium and the same final process time $t_{f}$, our inverse engineering protocol involves higher maximum velocities but the crane ends with much lower energy, almost ending in equilibrium. In the dotted part of the curves the limit of 2 m/s is surpassed.

## 6. Conclusions

We have applied an invariant based inverse engineering STA method to design fast trolley trajectories of a double pendulum overhead crane. In the small oscillations regime these trajectories guarantee that the transport does not induce any energy excitation, regardless of the initial condition of the double pendulum. We have first found the normal modes and from them the dynamical invariants. Using these invariants, it is possible to inverse engineer STA trolley trajectories. We have performed the numerical simulations with the exact dynamics to see the parameter intervals where the protocol is accurate. Comparisons are also made with less sophisticated trolley trajectories that demonstrate the advantage of the STA approach. We have worked out a particularly simple design for the trolley speed with three sine terms, (31). It should be clear that we have not really optimized the trolley trajectory. One of the interesting facts about STA methods is that the solutions to the inverse problem are not unique. That means that there is much room for finding specific trajectories that optimize variables of interest, or are robust with respect to specific perturbations or parametric uncertainties [35]. STA combine well in particular with optimal control theory [3]. Thus STA provide a useful avenue to minimize the sensitivity to parameter uncertainties, one of the weak points of open-loop approaches. Other possible extension of this work may be to tackle combined or sequential operations with transport and hoisting [25].

Compared to previous work on methods without feedback [26,28,36], this paper exemplifies and introduces the use of shortcuts-to-adiabaticity in mechatronics for multimode systems. We refrain from performing a numerical comparison with “input shaping” methods because virtually any result would be possible given the flexibility of both input-shaping and STA methods to accomodate a vast family of possible designs for the trolley motion, corrections for increased robustness with respect to parameter uncertainties or noise. Nevertheless we would like to underline the simplicity of the basic invariant-based engineering for the moving double pendulum crane, compared to input-shaping approaches [26,28,36]. Even if the choice among methods may be a matter of taste and previous experience, we would like to argue that STA should be in the the toolbox of control methods, if only because STA are well tested and have been intensely developed theoretically along different approaches and applied to many experiments in AMO (atomic, molecular, and optical), and solid state physics [3]. Thus engineering applications may benefit from an important framework of techniques and concepts. By the way, a positive influence in the opposite direction, from mechatronics to AMO physics, is also expected. For example, state manipulation in AMO science has much to learn from a long experience on control with feedback in mechatronics.

## Appendix A. Simple Ansatz for Trolley Velocity

The ansatz with three free parameters (31),

(A1) $$\dot{x}\left(t\right)=\sum_{k=1}^{3}a_{k}\sin\frac{(2k-1)\pi t}{t_{f}},$$

is an even function with respect to $t=t_{f}/2$ and it automatically satisfies $\dot{x}\left(t_{b}\right)=0$. We now rewrite the auxiliary Equations (28) and (29) as

(A2) $$\begin{array}{ccc} {\ddot{\beta }}_{j}+\omega_{j}^{2}\beta_{j} & = & \dot{x} \end{array}$$

for j=1,2, $\alpha_{1}=\beta_{1}\sqrt{Mg/l_{1}}\cos\theta$, and $\alpha_{2}=-\beta_{2}\sqrt{Mg/l_{1}}\sin\theta$. These are the equations of a driven harmonic oscillator, where the trolley velocity plays the role of the external driving force. The new variables $\beta_{j}$ should satisfy the BC $\beta_{j}\left(t_{b}\right)={\dot{\beta }}_{j}\left(t_{b}\right)={\ddot{\beta }}_{j}\left(t_{b}\right)=0$, see (30).

The solutions with initial condition $\beta_{j}\left(0\right)={\dot{\beta }}_{j}\left(0\right)=0$ to the above equations (A2) can be written in a compact form using the complex function [37]

(A3) $$\begin{array}{ccc} z_{j}\left(t\right) & = & \omega_{j}\beta_{j}\left(t\right)+i{\dot{\beta }}_{j}\left(t\right)\\ & = & ie^{-i\omega_{j}t}\int_{0}^{t}\dot{x}\left(\tau \right)e^{i\omega_{j}\tau }d\tau . \end{array}$$

They also satisfy ${\ddot{\beta }}_{j}\left(0\right)=0$ because $\dot{x}\left(0\right)=0$, see (A2). If we now impose that the integral term in the expression above vanishes at $t=t_{f}$ for j=1,2, the final time BCs $\beta_{i}\left(t_{f}\right)={\dot{\beta }}_{i}\left(t_{f}\right)={\ddot{\beta }}_{i}\left(t_{f}\right)=0$ will be also satisfied. Note that since $\dot{x}\left(\tau \right)$ is an even function in the integration interval, we may rewrite the integral above as

$$\begin{array}{c} \int_{0}^{t_{f}}\dot{x}\left(\tau \right)e^{i\omega_{j}\tau }d\tau =e^{i\omega_{j}t_{f}/2}\int_{0}^{t_{f}}\dot{x}\left(\tau \right)\cos\left[\omega_{j}\left(\tau -\frac{t_{f}}{2}\right)\right]d\tau \end{array}$$

so that only the cosine part of the remaining exponential may contribute by symmetry.

We have therefore two integral constraints, but three parameters to determine in (A1). The third, and last, constraint comes from the fact that the crane trajectory ends at $x(t_{f})=d$,

(A4) $$\begin{array}{ccc} x\left(t_{f}\right)=\int_{0}^{t_{f}}\dot{x}\left(\tau \right)d\tau & = & d. \end{array}$$

The three conditions are therefore those summarized in (33).

In the steps just described the sixteen boundary conditions in (30) are satisfied. Let us count them explicitly: two for $\dot{x}\left(t_{b}\right)$, four for the initial conditions set for $\beta_{j}\left(0\right)$ and ${\dot{\beta }}_{j}\left(0\right)$; two more because ${\ddot{\beta }}_{j}\left(0\right)$ vanishes automatically due to $\dot{x}\left(0\right)=0$; four are satisfied for the $\beta_{j}$ and their first derivatives at $t_{f}$ when nullifying the integral; this implies two more, $\ddot{\beta_{j}}\left(t_{f}\right)=0$ using $\dot{x}\left(t_{f}\right)=0$; finally the last two correspond to the initial condition x(0)=0 and final condition $x(t_{f})=0$.

Equation (A3) is also useful to analyze possible unstable behaviour due to border effects. For the proposed ansatz for the trolley velocity (A1) the only discontinuity will be in the initial (and final) acceleration but it behaves nicely with $t_{f}$,

(A5) $$\begin{array}{ccc} \ddot{x}\left(0\right) & = & -\ddot{x}\left(t_{f}\right)=\frac{225d\pi^{6}}{2\omega_{1}^{2}\omega_{2}^{2}t_{f}^{6}}. \end{array}$$

(The effect of $\ddot{x}\left(0\right)$ on the $\beta_{j}$ as a boundary term is made evident by integrating (A3) twice by parts.) We tried other ansatzes, polynomials in particular, with a much worse behaviour and serious boundary-driven excitations because of periodic singularities of $\ddot{x}$ at the boundary times.

If a null acceleration is imposed at boundary times, a further term in (31) will be needed and a discontinuity in the fourth derivative of x(t) scaling as $t_{f}^{-8}$ will be observed. Every odd derivative of a series like (A1) is zero by construction, regardless of the number of terms. In general, imposing a null 2nth derivative of x(t) leads to a discontinuity in the (2n+2)th derivative, scaling as $t_{f}^{-(2n+6)}$, i. e.,

(A6) $$\begin{array}{c} x^{\left(2n\right)}\left(t_{b}\right)=0\to x^{\left(2n+2\right)}\left(t_{b}\right)\propto t_{f}^{-(2n+6)}. \end{array}$$

As for the potential occurrence of resonances because of matching of frequencies in (A3), our ansatz (A1) leads to very stable and simple $\beta_{j}$ forms without any resonant behaviour, in particular no dangerous denominators arise. Thus our trolley trajectory avoids resonances from both modes in a simple direct way, for other methods using multi-mode input shaping see References [26,28].
